# Construction of Soft Prep Cadaver Pericardiocentesis Training Model and Implementation Among Emergency Medicine Residents

**DOI:** 10.21980/J87930

**Published:** 2023-04-30

**Authors:** Kathryn Oskar, Dana Stearns

**Affiliations:** * Massachusetts General Hospital, Brigham & Women’s Hospital, Department of Emergency Medicine, Boston, MA; ^ Massachusetts General Hospital, Harvard Medical School, Department of Emergency Medicine & Surgery, Boston, MA

## Abstract

**Audience:**

This procedure training model is designed for all levels of emergency medicine residents.

**Background:**

Pericardiocentesis is a relatively uncommon but potentially life-saving procedure within the scope of Emergency Medicine practice. As such, the Accreditation Council for Graduate Medical Education (ACGME) designates its competency as a requirement within emergency medicine residency programs. Because of its relative rarity, simulation-based training is often utilized to fill the gaps in clinical experience during emergency medicine residency training. There have been several models of pericardiocentesis training, including gel-based models that can be purchased or constructed,^1^–^3^ non-gel models,^4^ and cadaveric models.^5^ In this paper, we describe the fabrication of a high-fidelity cadaveric model and report emergency medicine resident experience with this model. Training programs can use this model to increase trainee competence and confidence with this high-acuity, low-frequency procedure.

**Educational Objectives:**

By the end of this session, residents will gain increased procedural competence and confidence with pericardiocentesis. Residents will be able to identify necessary supplies for the procedure, identify relevant surface anatomy and ultrasound views, and successfully aspirate fluid from model effusion.

**Educational Methods:**

We created a pericardial effusion in a soft prep cadaver by placing a catheter into the pericardial sac and then infusing normal saline via intravenous fluid tubing. Learners were then able to practice aspiration of pericardial fluid via landmark and ultrasound-guided approaches under observation by facilitators able to offer real-time feedback.

**Research Methods:**

Learners were asked to complete a survey assessing pre-intervention and post-intervention subjective confidence in their ability to perform pericardiocentesis and were asked for qualitative feedback on the experience of using the training model.

**Results:**

All residents were able to successfully visualize the pericardial effusion and perform needle aspiration via parasternal and subxiphoid approaches under dynamic ultrasound guidance, allowing needle visualization. All residents reported a subjective increase in procedural confidence and competence after practicing with this training model.

**Discussion:**

Overall, the primary benefit of this training model cited by emergency medicine residents was that it closely approximates reality. This model is re-usable, relatively durable, and reproducible. Emergency medicine residencies associated with academic medical centers that already utilize cadavers for education may relatively easily incorporate this training model into their procedure training curriculum.

**Topics:**

Pericardiocentesis, simulation, task trainer.

## USER GUIDE

**Table t2-jetem-8-2-i8:** 

List of Resources:	
Abstract	8
User Guide	10

[Table t2-jetem-8-2-i8] **Learner Audience:**

Interns, Junior Residents, Senior Residents

**Time Required for Implementation:**

Creation of our pericardiocentesis model took approximately 30 minutes total.

Procedure practice was offered in one-hour workshops. Participants were not required to stay for the entirety of the hour. The first five minutes of this time was spent reviewing the procedure technique and necessary supplies. For the rest of the hour, residents were permitted as many pericardial aspiration attempts as desired under direct observation and real-time feedback from facilitators.

**Recommended Number of Learners per Instructor:**

One instructor to 2–5 learners

**Topics:**

Pericardiocentesis, simulation, task trainer.

**Objectives:**

By the end of this activity, learners will be able to:

1. State the required supplies for performing landmark and ultrasound-guided pericardiocentesis procedure.
2. Identify surface anatomy landmarks and obtain ultrasound views required for pericardiocentesis.
3. Describe all critical actions to perform pericardiocentesis.
4. Successfully aspirate fluid from simulated pericardial effusion using a subxiphoid, landmark-based approach.
5. Successfully aspirate fluid from simulated pericardial effusion using dynamic ultrasound-guided needle visualization from both subxiphoid and parasternal approaches.

### Linked objectives and methods

We attempted to make this procedure training model as realistic as possible so that the mental steps and procedural skills gained by using the training model would be more easily translated to actual patient care and resuscitation. Before making a puncture attempt, participants are expected to familiarize themselves with and verbalize each piece of required equipment (objective 1). Next, participants were asked to identify key surface landmarks on the cadaver and demonstrate the ability to obtain an appropriate ultrasound view of the simulated effusion (objective 2). Aspiration attempts were monitored by the session instructors who were able to offer verbal corrections until participants successfully aspirated fluid using landmark-based subxiphoid, ultrasound-guided subxiphoid, and ultrasound-guided parasternal long approaches (objectives 4 and 5). While making an aspiration attempt, participants were reminded to verbalize their actions including needle angle of the approach, visualization of the needle when using dynamic ultrasound guidance, and constant aspiration during needle advancement. After successful aspiration of pericardial effusion, participants were asked to demonstrate another mental rehearsal of the procedure by describing all steps of the procedure from beginning to end, and the key structures at risk of damage based on approach (objective 3).

### Recommended pre-reading for instructor

- Nickson C. Pericardiocentesis. Life in the Fast Lane. Reviewed and revised 4 May 2014. Accessed Nov 3, 2020. Available at: https://litfl.com/pericardiocentesis
- Episode 54 – The Pericardium. FOAMcast: An Emergency Medicine Podcast. Aug 8, 2016. Accessed March 10, 2023. Available at: https://foamcast.org/2016/08/08/episode-54-thepericardium
- Farkas J. Pericardial tamponade. Internet Book of Critical Care (IBCC). Nov 10, 2021. Accessed March 10, 2023. Available at: https://emcrit.org/ibcc/tamponade
- Donaldson R. Pericardiocentesis. WikEM. April 12, 2022. Accessed March 10 2023. Available at: https://wikem.org/wiki/Pericardiocentesis
- Fitch MT, Nicks BA, Pariyadath M, McGinnis HD, Manthey DE. Emergency pericardiocentesis. *N Engl J*
- *Med*. 2012;366(12):e17. Accessed March 10, 2023. Available at https://www.nejm.org/doi/10.1056/NEJMvcm0907841

### Implementation Methods

Emergency medicine resident participants were introduced to the training model via instructor demonstration of surface anatomy landmarks, probe placement to acquire appropriate ultrasound windows, and technique for manipulating the syringe with a needle to perform pericardial aspiration. Participants were then asked to identify relevant surface anatomy and cardiac structures, including the simulated effusion on point-of-care ultrasound to reinforce the instructor's demonstration.

Participants next practiced accessing the effusion using direct needle visualization under dynamic ultrasound guidance. A 20-gauge spinal needle and 10cc syringe was used for aspiration. A small caliber needle was selected to minimize damage to the pericardium and prolong the usability of the model. Real-time feedback was offered by two instructors, a senior emergency medicine resident and an emergency medicine attending who had created the model.

Successful performance of the procedure was qualified as visualization of the needle tip in the pericardial effusion on ultrasound and the presence of clear fluid in the syringe. Residents were allowed as many attempts at pericardial aspiration as desired. Participants successfully performed both parasternal and subxiphoid ultrasound-guided approaches. The placement of the catheter to infuse fluids into the pericardial space at the cardiac apex, unfortunately, limited the ability to practice an ultrasound-guided apical approach to aspiration of the pericardial effusion. Finally, participants were asked to practice using a landmark-guided subxiphoid approach without ultrasound guidance. Similarly, real-time feedback was given as residents practiced, and success was qualified as the presence of clear fluid in the syringe.

The total number of punctures made in the model during creation and resident training was not recorded. Each resident was expected to complete at least three punctures (an ultrasound-guided parasternal long approach, an ultrasound-guided subxiphoid approach, and a landmark-based subxiphoid approach), and oftentimes elected to practice several additional punctures. The model demonstrated no breakdown after an estimated 40 to 50 punctures including all punctures made to test and demonstrate use of the model, plus the punctures made by residents during the training session. The exact number of punctures at which point the model tissue starts to break down has not yet been discovered. Due to the nature of the soft prep cadaver, prior puncture sites did not leave obvious permanent markings on the superficial tissue.

### List of items required to replicate this innovation

1. Soft prep cadaver
2. Point of care ultrasound machine with phased array probe (Phillips Lumify was used to create this model)
3. Ultrasound gel
4. #10 scalpel
5. Kelly clamp or any preferred instrument for blunt dissection
6. Weitlaner retractor (not essential to create the model but helpful for better visualizing pericardial sac in small chest wall incision)
7. Toothed forceps
8. Teleflex Medical Arrow Arterial Catheter Kits - Arterial Catheter Kit, 18G × 4–1/4”
9. Intravenous fluid tubing
10. At least one 1-liter bag of normal saline
11. Pressure bag
12. Sutures & needle driver
13. 18- or 20-gauge spinal needle
14. 10 or 20mL syringe

### Approximate cost of items to create this innovation

The pre-existing resources facilitated the creation of this model at the authors’ affiliated medical school, which had already used soft-prep cadavers, surgical instruments, and portable ultrasound machines for pre-existing courses. These resources were generously made available free of cost to create our model. Other required supplies, including intravenous tubing, catheters, and pressure bags were obtained from the emergency department when supplies were inadvertently opened for patient care but not used. This system of supply recycling reduces overall medical waste. It decreases the cost of creating this model, since all supplies were free of cost but may initially create a less reliable stream of supplies.

### Detailed methods to construct this innovation

**Figure 1:**
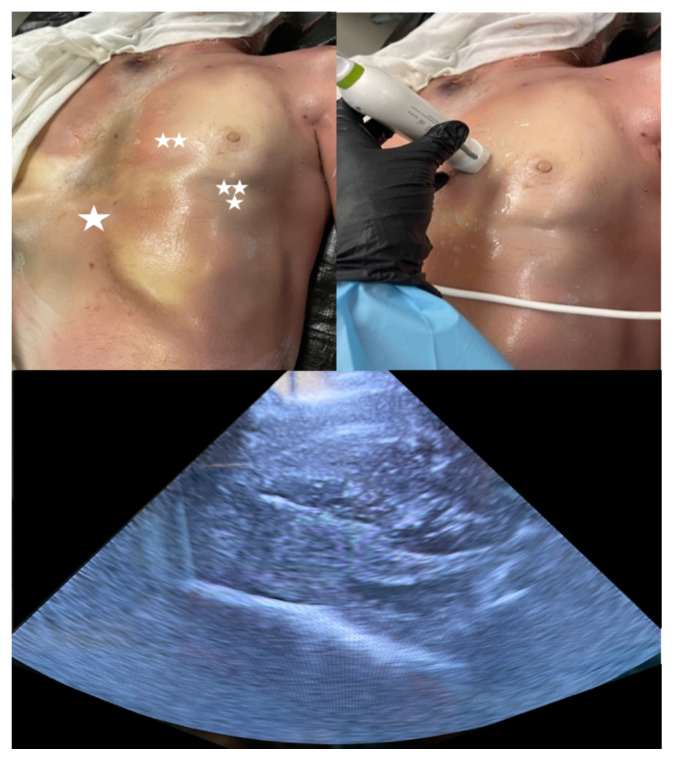
Soft prep cadaver Top left: anatomical location for subxiphoid (one star), parasternal (two stars) and apical (three stars) approaches. Top right: using ultrasound to demonstrate parasternal view. Bottom: parasternal view prior to creation of effusion

**Figure 2:**
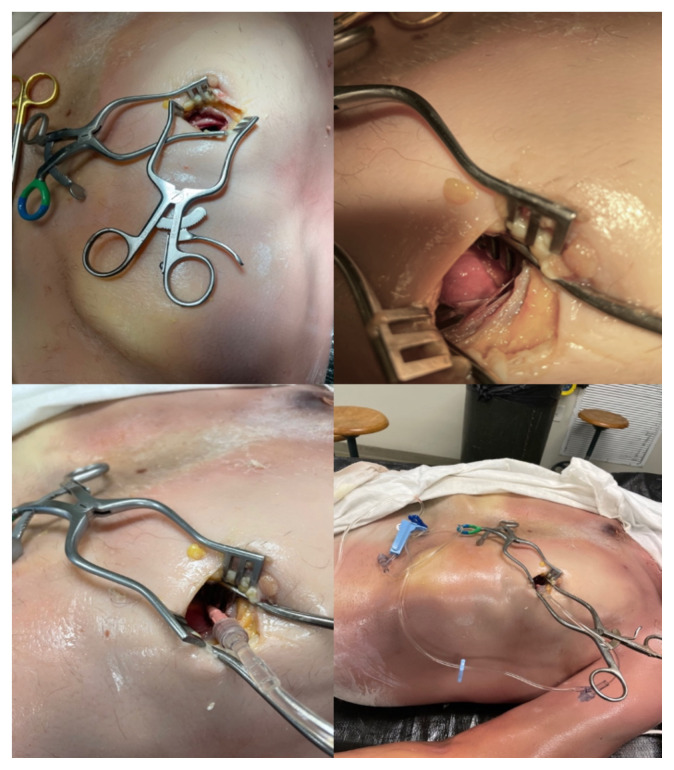
Creation of model effusion Top left: small thoracotomy incision made directly over the cardiac apex Top right: magnified view of anatomy including lung and epicardial fat. Pericardial sac is deep to these structures. Bottom left: catheter inserted into pericardial sac Bottom right: infusing fluid using tubing and catheter into pericardial space.

**Figure 3:**
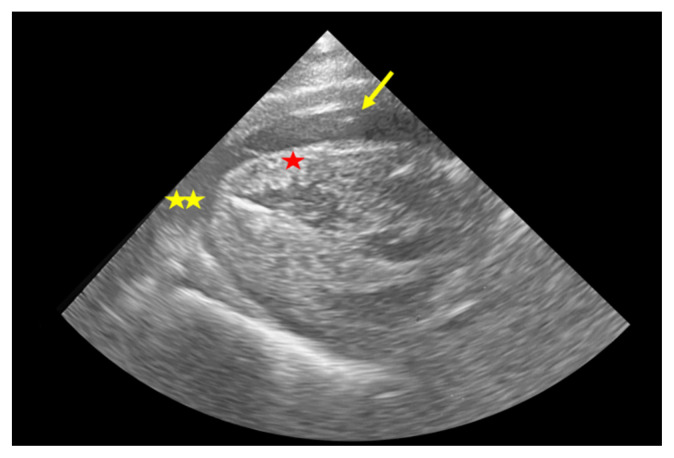
Parasternal long view of model effusion The myocardium (red star) and pericardial effusion (yellow stars) are easily visualized. Here a needle tip (arrow) can be seen aspirating the anterior pocket.

1. The soft prep cadaver Donor was prepared using the soft-prep solution: So Soft, Trinity Fluids, LLC Canton, MI; Ethanol 35%, Ethylene Glycol 35%, Formaldehyde 1%, Phenol 1%, and the balance was water; for this project’s purposes, the solution was infused undiluted. The Donor was stored at 45 degrees Fahrenheit; it was then used at 65 degrees Fahrenheit in a ventilated environment within the affiliated medical school anatomy laboratories.
2. To create the pericardial effusion model, we first identified the cardiac apex on the cadaver using ultrasound visualization to make a small, precise incision on the overlaying chest wall (Figure 1).
3. Using a #10 scalpel, a 3–4 centimeter incision was made in the cadaver’s anterior chest wall just over the cardiac apex. Blunt dissection was performed through the left 5^th^ intercostal space. The left lung was identified and retracted superiorly and laterally until the pericardial sac and left phrenic nerve was encountered.
4. Using toothless forceps, the parietal pericardium lying anterior to the phrenic nerve was tented and traction was held on the tissue while an 18-gauge, 4 ¼-inch angiocatheter needle was used to pierce the pericardial sac.
5. The catheter was advanced fully into the pericardial space and the needle removed.
6. The heart and left lung positions were restored with the catheter in place. The catheter was then sutured in place to the intercostal soft tissues, with the female end exposed at the skin surface.
7. Intravenous tubing was then attached to the angiocatheter and normal saline was infused with a pressure bag (Figure 2).
8. The subcutaneous tissues and skin were closed with sutures to stabilize the angiocath and to better approximate an intact chest wall.
9. Using a pressure bag, normal saline was infused into the pericardial space. Pressure was maintained or increased as needed to maintain a large enough effusion to aspirate.
10. After saline was infused through the tubing and catheter, ultrasound visualization was used to visualize the heart and confirm the presence of simulated pericardial effusion (Figure 3).

### Results and tips for successful implementation

**Figure 4:**
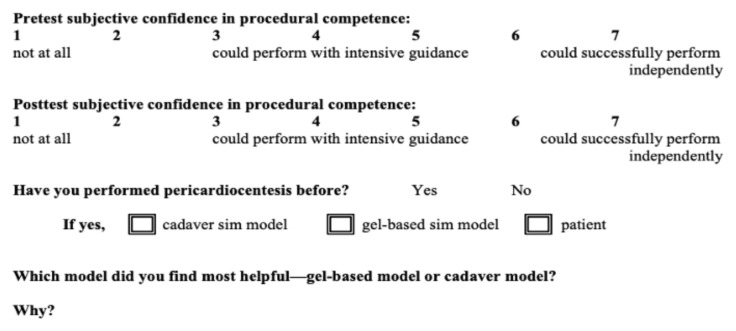
Survey administered to participating emergency medicine residents

This simulation model was offered for resident practice as part of an informal series of anatomy lab procedure practice sessions. Nine emergency medicine residents of various levels of training (first-year through third-year) volunteered to trial this model. After practicing with the model, a survey was administered asking participants to rank subjective pre- and post-intervention procedural confidence on a modified Likert scale from 1—not confident at all—to 7—could successfully perform independently. Resident participants were also asked to describe previous experience with performing pericardiocentesis and were asked for qualitative feedback on the experience of practicing with our model (Figure 4). As an educational session that was part of the training curriculum, this was exempt from Institutional Review Board review.

All residents were able to successfully visualize the pericardial effusion and perform needle aspiration via parasternal and subxiphoid approaches under dynamic ultrasound guidance allowing needle visualization. All residents reported a subjective increase in procedural confidence and competence after practicing with this training model. Out of a maximum score of 7, average pretest confidence was 2.1 + 0.9, average posttest confidence 5.3 + 0.6, and an absolute change of +3.2 (95% CI 2.2 – 4.3, p <0.001). Qualitative feedback on the practice model highlighted the positive impacts of the realistic nature of the training model (Table 1).

This model would optimally be incorporated into a formal curriculum for rare procedure practice that involves pre-reading or videos, one practice session with real-time feedback, and then a follow-up formal assessment of competence using a mastery-based learning assessment.

## Figures and Tables

**Table 1:** Participant Survey Results

Participant Number	Pre-test subjective confidence	Post-test subjective confidence	Prior experience with pericardiocentesis?	Which model is helpful & why
1	3	6	No	Cadaver model - loved how realistic this was, very useful
2	1	4	No	Cadaver is best
3	1	4	No	Extremely well done & valuable session
4	4	6	No	Cadaver model – realistic
5	1	6	No	Cadaver model – most realistic & good for feel/sensation of going through tissue
6	1	5	No	Using the cadavers gave me a sense of landmarks and the realistic challenges I might face at bedside (vacuum needed to pull back syringe, syringe occluding, angle of insertion vs angle of ultrasound to better visualize the needle, etc). So helpful.
7	1	5	No	Have only done this cadaver model but found it very helpful, better than usual gel models.
8	4	6	Yes – patient	Cadaver gave incredibly realistic views
9	3	6	No	Realistic anatomy and ultrasound guidance
